# Beyond the First Trimester: Social Determinants of Delayed Prenatal Care at a Community Health Center Using the PRAPARE Tool

**DOI:** 10.1007/s40615-024-02052-7

**Published:** 2024-06-27

**Authors:** Abbie Page, Rebecca McCann, Sarah Maness, Maya Merriweather, Page D. Dobbs

**Affiliations:** 1 Department of Health, Human Performance and Recreation, University of Arkansas, 346 West Ave. Suite 317, Fayetteville, AR 72701 USA; 2 St. Francis House NWA Inc. dba. Community Clinic, Springdale, AR 72764 USA; 3 Center for Public Health and Technology, University of Arkansas, 346 West Ave. Suite 317, Fayetteville, AR 72701 USA; 4 Eleanor Mann School of Nursing, University of Arkansas, Fayetteville, 72701 AR USA; 5 Department of Health Education and Promotion, East Carolina University, Greensville, NC 27858-4353 USA; 6 Fay W. Boozman College of Public Health, University of Arkansas for Medical Science, Little Rock, AR 72205 USA; 7 Obstetrics and Gynecology, Indiana University School of Medicine, Indianapolis, IN 46202 USA

**Keywords:** PRAPARE, Social Determinants of Health, Community Health Center, Prenatal Care

## Abstract

Social determinants of health have been used to explore associations with pregnancy outcomes and the birth weight of infants; however, research employing individually based social risk measures has not examined associations among underserved populations, including pregnant persons at community health centers. Data were collected from a sample (*n* = 345) of pregnant persons who sought care at a community health center between January 2019 and December 2020. Social risks of pregnant patients were measured using the PRAPARE tool. First, associations between patients’ social risks and trimester in which they initiated care were assessed using ANOVAs, grouping social risk by PRAPARE social determinant domains (persona characteristics, family and home, money and resources, and social and emotional health). ANOVAs were stratified by ethnicity. Next, a multivariate logistic regression examined associations between social measures and seeking care after the first trimester. Patients who sought care in the first trimester reported more financial needs than those who sought care in the second (*p* = .02) or the third (*p* = .049). Hispanic patients who sought care in the first trimester reported more monetary needs than those who sought care in the second trimester (*p* = .048), and non-Hispanic patients who sought care in the first trimester reported greater family and home needs than those who sought care in the second trimester (*p* = .47). Those who experienced stress were 3.07 times as likely to seek care after the first trimester as those who reported no stress. CHC may reduce social risk among poor and underserved communities by reducing barriers to access to care.

## Introduction

 The health of an overall population is sensitively measured by infant morbidity, low birth weight, and infant mortality [1]. Compared to other developed nations, the USA has some of the worst perinatal outcomes worldwide [2], with 1 in 12 live births (8.3%) reported to be low birth weight infants in 2019 [3]. The prevalence and incidence of pregnancy and birth-related health issues can be complex and multifactorial as a pregnancy is impacted by physiologic, socio-economic, and behavioral factors [4].

Research suggests Social Determinants of Health (SDoH) can influence pregnancy outcomes and birth weight of infants [5, 6]. SDoH are non-medical factors that can have both positive and negative impacts on the health of an individual and may have greater impact on health than healthcare or lifestyle, making them important to assess and address [7]. The World Health Organization defines SDoH as “conditions in which people are born, grow, work, live and age and the wider set of forces and systems shaping the conditions of daily life” (para. 1) [7]. SDoH have been used to help explain health inequities by determining health disparities between different groups of people [8]. For example, increasing access to healthcare and technology can benefit patients who may not otherwise receive it due to social factors [9]. Many SDoH are rooted in systemic racism, which unequally impact people of racial and ethnic minority groups. Exploring social risks can contribute to reducing the large gaps in health outcomes between racial and ethnic minority groups in the USA [10]. Some examples of SDoH that have been found to be associated with negative pregnancy outcomes include socio-economic status, race/ethnicity, and educational attainment. Research suggests that low socio-economic status is associated with teen pregnancy, access to prenatal care, and inadequate prenatal care, all of which are predictors of preterm birth and low birth weight [6, 11–13]. Further, racial inequities have been found to significantly influence pregnancy outcomes. For example, non-Hispanic Blacks are twice as likely to have low birth weight infants as non-Hispanic whites [14], and while infant mortality rates have been declining, non-Hispanic Blacks continue to experience infant mortality rates significantly higher than non-Hispanic whites that are driven by racial inequities before any other social determinant of health [15]. In addition, there are variations on initiation of prenatal care among racial groups; non-Hispanic whites are more likely to initiate prenatal care in their first trimester than other racial/ethnicity groups, including non-Hispanic Native Hawaiian Pacific Islander (NHPI) [16]. Those with lower educational attainment have been found to have late or no prenatal care and increased odds of delivering a low-birth-weight infant [5, 13, 16]. Although social risk factors are not the only variables driving poor prenatal outcomes, which include racism-related stress and experiences of direct racism, the role of SDoH is important to explore to improve equity in pregnancy outcomes [17, 18].

Community Health Centers (CHCs) play an integral role in providing quality and cost-effective healthcare to underserved patients who may not otherwise be able to afford it [19, 20]. Since 1965, these CHCs have reduced barriers to care such as cost, insurance status, distance, and language [21]. As a Federally Qualified Health Center, CHCs require reporting to the Uniform Data System which creates a unique opportunity for researchers to access real-time data on patient access to prenatal care by trimester [22]. Having access to a CHC increases a community’s utilization of healthcare services, including prenatal care, especially in low-income and racial minority populations [23, 24]. Furthermore, reducing or eliminating barriers to healthcare can improve health outcomes for expectant mothers and their children [23].

Research has used instruments to explore combined SDoH among patients from CHCs [25–27]. One instrument used to measure SDoH is the Protocol for Responding to and Assessing Patients’ Assets, Risks, and Experiences (PRAPARE) Tool, developed by the National Association of Community Health Centers. This instrument is widely used in CHCs across the USA to measure individual-level SDoH among patients by creating a combined score of social risks [27, 28]. The PRAPARE tool analyzes the following core measures: personal characteristics, family and home, money and resources, social and emotional health, other voluntary measures in PRAPARE (incarceration history, refugee status, safety, domestic violence), to identify social risk factors [28]. The PRAPARE instrument is integrated into many CHCs electronic medical records which allows it to generate a risk score after the provider or the patient completes the necessary fields. With the use of social risk stratification tools such as the PRAPARE tool, CHCs can justifiably determine a patient’s social risk factors and how they may affect their overall health outcomes. To date, research has used the PRAPARE to examine COVID testing and positivity rates [25], social risk among pregnant patients seeking prenatal care at a CHC [26], and social risk and SDoH barriers among a clinical population [27]. Despite research that has explored the impact of individual SDoH on pregnancy and birth outcomes [11], there remains a gap in research regarding the relationship between SDoH, particularly using the PRAPARE tool, and initiating a prenatal exam. The purpose of this study is to explore the relationship between social risk of pregnant persons and the onset of their initial prenatal exam.

## Methods

Data used in this study were collected between January 2019 and December 2020 from a single CHC in the Midwest United States. This retrospective, cross-sectional analysis exclusively uses information collected from patient Electronic Medical Records (EMR), using eClinical Works, including International Classification of Disease (ICD-10) coding, and Current Procedural Terminology (CPT) coding. EMR data was stored on protected servers by the CHC. The first author works on the CHC and was able to securely request data based on items included in the current study.

**Fig. 1:**
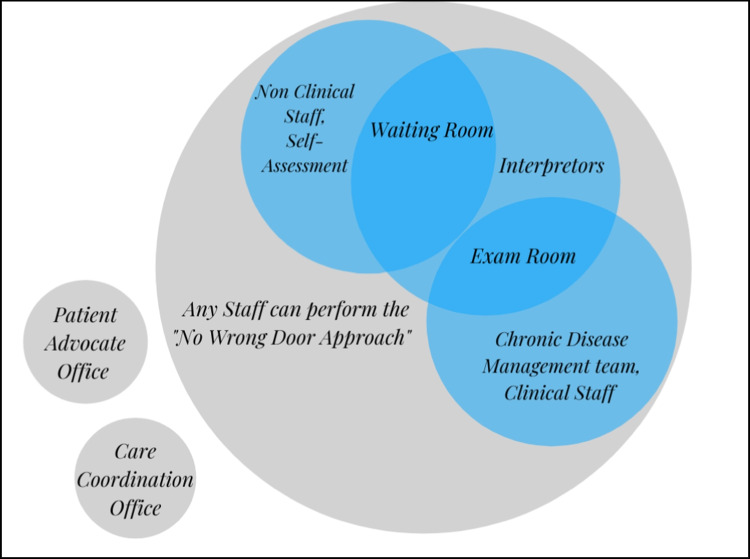
Completion of PRAPARE Items at a community health center

Consent was obtained during the patient intake process via policies that comply with the Health Insurance Portability and Accountability Act (HIPAA). Due to HIPAA restrictions, we were unable to obtain information about those who did not provide consent. Data were collected using a “no-wrong door” approach, where participant information were imputed into the EMR by the patient during their intake process or by staff throughout the care continuum (e.g., interpreters assisting patients in the waiting room and exam rooms, staff assessing patient needs during their visit). The PRAPARE tool is currently offered in 25 languages to ensure cultural validation for a wide variety of populations with translated versions provided at prepare.org. This information was collected through motivational interviews, paper surveys, and general conversation with any CHC staff. See Fig. 1 for a visual description of this approach. All data used in this study were de-identified and met HIPAA standards, and the current study was exempt from review by the [Blinded for Review] Institutional Review Board. Ethical standards of HIPAA ensure that only data from consenting patients was confidentially included in the current study.

### Participants

The CHC where data were collected provides prenatal care up to 28 weeks of gestation, offering referral to a local OB-GYN for delivery at the third trimester. Earlier transfer of care takes place in the case of high-risk needs, such as in pregnancies with multiple gestation. EMA data from all consenting patients seen for prenatal care during the indicated period were selected for the current study (*n* = 1463); however, data were delimited to only those confirmed pregnant during the evaluation period who fully completed the PRAPARE tool to generate a PRAPARE score (*n* = 345). EMA data from a prenatal visit, without a confirmed pregnancy test, and without all items of the PRAPARE tool completed were excluded from the study (*n* = 1118).

### Measures

Using the EMR, patient demographic information was pulled to reflect patient age, race, ethnicity, preferred language, and poverty level. Federally Poverty Level was calculated using a smart form in the EMR that calculates self-reported household income and size, based on current Federally Poverty Level issued by the Department of Health and Human Services [29]. Due to the small sample size, all demographic measures were dichotomized.

With possible scores ranging from 0 to 22 to indicate a level of relative social risk, the PRAPARE tool can measure a patient’s risk directly from the information input in the clinic EMR. The PRAPARE tool is made up of 15 items, all of which produce a single point score, except one item which can give up to seven points. Details on this tool and each measure have been cited elsewhere [25, 28]. Each item belongs to a single domain under the PRAPARE tool: Personal Characteristics, Family and Home, Finances and Resources, and Emotional Health. Patient responses were dichotomized to reflect the differences between those who score on each item (1) and those who do not score on the item (0). All PRAPARE items came from the national PRAPARE social determinants of health assessment protocol, developed, and owned by the National Association of Community Health Centers, in partnership with the Association of Asian Pacific Community Health Organization, the Oregon Primary Care Association, and the Institute for Alternative Futures. For more information, visit www.nachc.org/prapare. Initial prenatal visit dates were collected from the EMR using CPT coding, corresponding with the same date as the confirmation of pregnancy reflected in the ICD-10 code.

### Data Analysis

Social risk was measured by calculating the average PRAPARE scores (M = 6.3) and using each standard deviation (SD = 2.4) as greater risk (scores 0–4, no risk; scores 4–5, low risk; scores 6–7, moderate risk; and scores 8 + high risk) [28]. Using chi-square analyses, we examined for differences in risk score between reported demographic variables. We then summed individual item scores within respective domains (Personal Characteristics, Family and Home, Finances and Resources, and Emotional Health) to create an overall domain score. Differences between PRAPARE scores of those who sought initial care in the first, second, and third trimester were compared for each domain score using a one-way ANOVA. Lastly, a logistic regression was used to explore the association between each PRAPARE item and seeking care after the first trimester. Adjusted odds ratio (aOR) and 95% Confidence Intervals (95% CI) were used to report findings from the regressions. Unreported values excluded responses from analyses in the regression model. Statistical significance was evaluated a priori based on an α of 0.05. All analyses were conducted using IBM SPSS 27 software [30]. 

## Results

### Participant Characteristics

Overall, 61.2% (*n* = 211) of the patient sample reported moderate or high social risk scores (indicating 6 or more unmet needs) based on the PRAPARE tool. The average age of women in this sample (*n* = 345) was 28.1 years (SD = 6.5). Overall, 62% of the sample identified as Hispanic or Latino. The largest racial group within the sample was White (44.6%). Of the patient sample, 45.5% preferred receiving care in English (45.5%), while the rest preferred receiving care in Spanish (47.2%) or Marshallese (7.2%). Most (92.7%) of the sample was living at 200% or greater of the Federal Poverty Level. There were significant associations between characteristics of those at and above low social risk for age (*p* < .01), ethnicity (*p* < .001), race (*p* < .001), language preferred for care (*p* < .001), and federal poverty level (*p* < .001). See Table 1.

**Table 1:** Descriptive statistics by social risk score (*N* = 345)

	Full sample		Low risk		> Low risk		*p*-value
	*N* = 345		*N* = 134		*N* = 211		
	*n*	%	*n*	%	*n*	%	
Age							
15–19	255	73.9	109	81.3	146	69.2	0.009
20–29	23	6.7	10	7.5	13	6.2	
30–39	67	19.4	15	11.2	52	24.6	
Ethnicity							
Hispanic/Latino	119	34.5	70	52.2	49	23.2	0.001
Non-Hispanic/Latino	214	62.0	58	43.3	156	73.9	
Unknown	12	3.5	6	4.5	6	2.8	
Race							
Caucasian	154	44.6	71	53.0	83	39.3	0.001
NHPI college	44	12.8	21	15.7	23	10.9	
American Indian/Asian/Black	21	6.1	12	9.0	9	4.3	
Unreported	126	36.5	30	22.4	96	45.5	
Language							
English	157	45.5	95	70.9	62	29.4	0.001
Spanish	163	47.2	30	22.4	133	63.0	
Marshallese	25	7.21	9	6.7	16	7.6	
Federal Poverty Level							
>200% FPL	305	92.7	109	86.5	196	96.6	0.001
<200% FPL	24	7.3	17	13.5	7	3.4	

**Table 2:** Differences between SDoHs by initiation of prenatal care

SDoH domain	First trimester *N* = 49	Second trimester *N* = 181	Third trimester *N* = 27	* F* value
All participants	*M (SD)*	*M (SD)*	*M (SD)*	
Personal characteristics (0–3)	1.31 (0.80)	1.36 (0.78)	1.30 (0.91)	0.17
Family and home (0–2)	0.29 (0.50)	0.24 (0.47)	0.30 (0.47)	0.26
Money and resources (0–11)	3.91 (2.53) ^a^	3.02 (1.89)^a^	2.74 (1.38) ^b^	4.65*
Social and emotional health (0–2)	1.16 (0.69)	1.20 (0.80)	1.30 (0.69)	0.27
Hispanic ethnicity only	*n* = 33	*n* = 104	*n* = 17	
Personal characteristics	1.70 (0.47)	1.76 (0.45)	1.88 (0.33)	0.98
Family and home	0.18 (0.39)	0.23 (0.49)	0.35 (0.49)	0.75
Money and resources	3.88 (1.11) ^a^	3.36 (1.00) ^a^	3.29 (1.16)	3.36*
Social and emotional health	1.15 (0.71)	1.25 (0.81)	1.35 (0.70)	0.40
Non-Hispanic ethnicity only	*n* = 13	*n* = 70	*n* = 10	
Personal characteristics	0.62 (0.77)	0.86 (0.79)	0.67 (0.21)	2.55
Family and home	0.62 (0.65) ^a^	0.26 (0.44) ^a^	0.20 (0.42)	3.42*
Money and resources	4.00 (1.41)	3.50 (1.25)	3.30 (1.25)	0.93
Social and emotional health	1.08 (0.64)	1.20 (0.63)	1.12 (0.73)	0.08

17 missing responses

^a^ Represents significant differences between first and second trimester.

^b^ Represents significant differences between first and third trimester

Scheffe’s post hoc analysis employed.

* Significant at 0.05

**Table 3:** SDoH items association with seeking prenatal care after the first trimester (*n* = 245)

		*N* %)		95% CI	
			aOR	Lower	Upper
Ethnicity					
Not Hispanic or Latino		92 (37.6)	1.00		
Hispanic or Latino		153 (62.4)	0.79	0.29	2.11
Language most comfortable speaking					
English		106 (43.3)	1.00		
Language other than English		139 (56.7)	1.35	0.60	3.01
Race					
White		200 (81.6)	1.00		
Race other than white		45 (18.4)	3.14	0.81	12.22
Housing situation					
I have housing		216 (88.2)	1.00		
I do not have housing		29 (11.8)	0.93	0.32	2.74
Worried about housing					
No		213 (86.9)	1.00		
Yes, worried about housing		32 (13.1)	1.26	0.42	3.81
Work situation					
Full time work		65 (26.5)	1.00		
Unemployed, part-time		180 (73.5)	0.87	0.37	2.02
Main insurance					
Private insurance		77 (31.4)	1.00		
Medicaid, medicare, state aid, breast care		168 (68.6)	1.04	0.49	2.21
Educational attainment					
More than a high school degree		60 (24.5)	1.00		
High school degree or less		185 (75.5)	1.20	0.52	2.80
Number of unmet needs					
No problem meeting needs		183 (74.7)	1.00		
Unable to get 1 + needs		62 (25.3)	0.33	0.15	0.75
Transportation issues					
No transportation issues		218 (89.0)	1.00		
Transportation kept from medical or non-medical meeting		27 (11.0)	0.27	0.10	0.72
Poverty level					
Above 200% poverty		20 (8.2)	1.00		
Below 200% poverty		225 (81.8)	0.28	0.03	2.46
Talk to people you care about					
More than 5 times a week		106 (43.3)	1.00		
Less than once a week to 5 times a week		139 (56.7)	0.58	0.27	1.23
Stressed					
Not at all		86 (35.1)	1.00		
A little bit to very much		159 (64.9)	3.07	1.37	6.90

### SDoH and Initiation of Prenatal Care

Among domains of SDoH included on the PRAPARE tool, we found money and resources to be the only area that was significantly associated with the trimester in which the pregnant patients sought initial care (Table 2). We found patients that sought initial care in the first trimester had significantly more financial needs (M = 3.91, SD = 2.53) than those who sought care in the second trimester (M = 3.02, SD = 1.89, *p* = .02) or the third trimester (M = 2.74, SD = 1.38, *p* = .049). When stratified by ethnicity, we found patients that Hispanic patients that sought initial care in the first trimester (M = 3.88, SD 1.11) had significantly more financial needs than patients who sought initial prenatal care in the second trimester (M = 3.36, SD = 1.00), *p* = .048). Among those who were not Hispanic, those with more family or home needs (M = 0.62, SD = 0.65) were more likely to see initial prenatal care in the first trimester than the second trimester (M = 0.26, SD = 0.44, *p* = .047).

When controlling for covariates included in the model, the number of unmet needs, transportation issues, and stress were all found to be associated with seeking initial prenatal care after the first trimester. Those unable to get one or more of the listed needs were less likely to seek initial prenatal care after the first trimester than those who reported no problem meeting their needs (aOR = 0.33, 95% CI = 0.15, 0.75). Also, those who had been unable to attend medical or non-medical appointments due to transportation issues were less likely to seek initial care after the first trimester than those with no transportation issues (aOR = 0.27, 95% CI = 0.10, 0.72). Finally, those who experienced any level of stress were three times as likely (95% CI = 1.37, 6.90) to seek initial prenatal care after the first trimester as those who reported no stress (Table 3).

## Discussion

The purpose of this study is to explore the relationship between social risk of pregnant persons and the trimester in which they sought initial prenatal care. Among this patient sample seeking care from a CHC, access to resources, reliable transportation, and stress were related to early initiation of prenatal care. More specifically and uniquely, we found that those with more unmet needs and greater issues accessing transportation were more likely to seek care in the first trimester, compared to their counterparts. While this finding was unexpected, it may provide insight into the trust developed between financially burdened populations and CHCs as a medical home.

While being diagnosed as pregnant can be stressful for some people [31], we found that stress was associated with increased odds of seeking care after the first trimester. Given the cross-sectional nature of the data, it is unclear if stress was elevated among pregnant women who sought care after the first trimester or if stress was simply indicated among women who sought care at the clinic, regardless of these circumstances. Women could have been stressed because they did not receive care earlier; however, pregnancy-related stress is well documented and can occur due to myriad reasons [32, 33]. Interventions that reduce the number of prenatal visits have reduced pregnancy-related stress [34]. In our study, most participants (73.9%) were women within the recommended age range for childbearing (20 to 34 years) [35]; thus, it may be feasible and beneficial to reduce the number of prenatal visits for women without health concerns in order to reduce stress among those who may have difficulty getting transportation to their appointments or taking time away from work for these visits.

By understanding the social needs of our patients, we are improving our knowledge about the patients that are served by the CHC, which is a strength of the study. Underserved patients are disproportionately affected by poor health outcomes, which is why CHCs serve this patient population with the goal of improving health equity. For example, a majority of the sample identified as Hispanic or Latino, which gives the opportunity to understand how to reduce gaps in health equity based on ethnicity. Knowing the predictor variables, like ethnicity, that may have an impact on the initiation of prenatal care, can help healthcare providers tailor their patient visits more specifically to these known variables. CHCs also provide resources to help mitigate SDoH barriers like transportation assistance [19]. Healthcare providers can address social needs brought up during the patient visit and ensure that the patient sees a patient advocate before leaving the clinic, so that their social needs are immediately addressed. Addressing social needs and the SDoH of the poor and underserved can have downstream effect on health outcomes and overall decision making that may improve the initiation of early prenatal care; with the potential to improve generational outcomes among this patient population.

## Limitations

This study is subject to limitations. Due to the cross-section method design, findings cannot establish causality. Also, delimiting factors such as a confirmation of pregnancy and a completed PRAPARE survey decreased the sample size substantially, resulting in 1118 patients not being eligible for the study. Although translators were used when needed and forms were translated in English and Spanish, expanding language translations may increase completion rates of the PRAPARE tool. Verbal translation may also bring concern regarding fear of stigma from patients in answering assessment questions. Lastly, self-reporting of information for the PRAPARE tool may be subject to reporting bias, reducing the reliability of the data. A strength of the study was the large Hispanic and NHPI populations. While this patient population may attract those with more social needs, this provides valuable data for exploring factors associated with access to healthcare among those with greater social needs than a private healthcare facility.

By creating a medical home for financially disadvantaged patients, CHCs may have generational effects by helping patients avoid delays in initiation of prenatal care. Healthcare providers at CHC are encouraged to educate their patients on the importance of prenatal care while also addressing any concerns and distress. Helping patients navigate social needs and life changes can reduce barriers to care and improve downstream health outcomes. Identifying social risk factors has benefits to prenatal health. By screening and assessing these risks, healthcare providers can tailor the patient care to meet the individual needs of the patient and community. CHCs serve a diverse population in communities, and through utilization of social risk assessment tools, such as the PRAPARE tool, they have additional resources to help identify health inequities experienced by the patient. Through early identification of social risk factors that this population experiences, healthcare workers can deploy more holistic interventions with the goal of improving health outcomes in their community.

## Data Availability

Data will be made available upon reasonable request.
